# Norepinephrine versus phenylephrine on cerebral tissue oxygen saturation during prophylactic infusion to prevent spinal hypotension for Caesarean birth

**DOI:** 10.1097/MD.0000000000037454

**Published:** 2024-03-08

**Authors:** Weiguo Wu, Qiang Zheng, Jinfeng Zhou, Xiujuan Li, Haipeng Zhou

**Affiliations:** a Department of Anaesthesiology, Qilu Hospital (Qingdao), Cheeloo College of Medicine, Shandong University, Qingdao, P. R. China.

**Keywords:** Caesarean birth, cerebral tissue oxygen saturation, norepinephrine, phenylephrine, spinal hypotension

## Abstract

**Background:**

Phenylephrine may cause a reduction in maternal cerebral tissue oxygen saturation (SctO_2_) during Caesarean birth to prevent spinal hypotension; however, the effect of norepinephrine has not been assessed. We hypothesized that norepinephrine was more effective than phenylephrine in maintaining SctO_2_ when preventing spinal hypotension during Caesarean birth.

**Methods:**

We conducted a randomized, double-blind, controlled study. Sixty patients were randomly assigned to prophylactic norepinephrine or phenylephrine to maintain blood pressure during spinal anesthesia for Caesarean birth. SctO_2_, systolic blood pressure, and heart rate were recorded. The primary outcome was the incidence of a 10% reduction of intraoperative SctO_2_ from baseline or more during Caesarean birth.

**Results:**

The norepinephrine group had a lower incidence of more than 10% reduction of intraoperative SctO_2_ from baseline than that of the phenylephrine group (13.3% vs 40.0%, *P* = .02). The change in SctO_2_ after 5 minutes of norepinephrine infusion was higher than that after phenylephrine infusion (−3.4 ± 4.7 vs −6.2 ± 5.6, *P* = .04). The change in SctO_2_ after 10 minutes of norepinephrine infusion was higher than that after phenylephrine infusion (−2.5 ± 4.4 vs −5.4 ± 4.6, *P* = .006). The norepinephrine group showed greater left- and right-SctO_2_ values than the phenylephrine group at 5 to 10 minutes. However, the change in systolic blood pressure was comparable between the 2 groups.

**Conclusion:**

Norepinephrine was more effective than phenylephrine in maintaining SctO_2_ when preventing spinal hypotension during Caesarean birth. However, the changes in clinical outcomes caused by differences in SctO_2_ between the 2 medications warrant further studies.

## 1. Introduction

Spinal hypotension is a common complication during Caesarean birth, with an incidence of up to 70% to 80%.^[1]^ Persistent hypotension can lead to maternal dizziness, nausea and vomiting, and fetal acidosis.^[2,3]^ Phenylephrine is recommended for preventing spinal hypotension during Caesarean birth.^[4]^ However, phenylephrine is commonly related to reductions in heart rate (HR) and cardiac output (CO).^[5]^ In 2015, the first randomized controlled trial in which norepinephrine was used for maintaining blood pressure in obstetric anesthesia found that norepinephrine could be an alternative to phenylephrine.^[6]^

Near-infrared spectroscopy (NIRS) provides continuous, noninvasive monitoring of regional tissue oxygen saturation and helps monitor adequate perfusion of different organs and peripheral tissues.^[7–9]^ Foss et al^[10]^ showed that, on average, phenylephrine may cause a reduction in maternal cerebral tissue oxygen saturation (SctO_2_) of nearly 9% from baseline during Caesarean birth when administered for preventing spinal hypotension; however, to the best of our knowledge, no such assessment of norepinephrine is available. SctO_2_ is closely associated with CO,^[11,12]^ and norepinephrine is more effective than phenylephrine in maintaining CO during Caesarean delivery.^[6]^ A Bayesian network meta-analysis about different vasopressors treating intraoperative hypotension patients during general/spinal anesthesia suggested that phenylephrine may decrease SctO_2_ compared with the other vasopressors.^[13]^ Therefore, we hypothesized that norepinephrine was more effective than phenylephrine in maintaining SctO_2_ when preventing spinal hypotension during Caesarean birth. This study aimed to compare the changes in maternal SctO_2_ between norepinephrine and phenylephrine administration to prevent hypotension during Caesarean birth.

## 2. Methods

Ethical approval was obtained from the Ethics Committee of Qilu Hospital of Shandong University in Qingdao, China (KYLL2021-014) before the trial started. Both verbal and written consent were obtained from all trial participants. This was a randomized, double-blind, controlled study conducted at Qilu Hospital (Qingdao) from October 2022 to April 2023. The study was registered before patient enrollment in the Chinese Clinical Trial Registry (registration no. ChiCTR2100047641; June 21, 2021).

Patients scheduled for elective Caesarean birth under spinal anesthesia were included in the study. Eligible participants were aged between 18 and 45, had American Society of Anaesthesiologists physical status 1 to 2, and had a full-term singleton pregnancy. Exclusion criteria included a history of traumatic cranial injury or cranial surgery, hypertension, coagulation abnormalities, cardiovascular disease, preoperative hemoglobin level <90 g/L, height <150 cm or ≥180 cm, weight <40 kg or ≥110 kg, and fetal abnormalities. This study adhered to the Consolidated Standards of Reporting Trials 2010 guidelines.

A graded dose-response study conducted by Ngan Kee et al^[14]^ estimated that the relative potency of norepinephrine:phenylephrine was 13.1:1.0 when administered in obstetric patients with postspinal hypotension. Accordingly, our study equated 8 μg of norepinephrine to 100 μg of phenylephrine. A random allocation sequence was prepared and the patient’s codes were successively placed into sealed and opaque envelopes. An investigator who was not involved in subsequent patient care or evaluation opened the sequentially numbered envelopes. The syringes used for continuous infusion were labeled as “study drug.” One 10-mL syringe used to treat hypotension was labeled as “rescue drug.”

The patients were routinely monitored after entering the operating room. HR, blood pressure using noninvasive intermittent oscillometric measurements, and pulse oximetry value were monitored (BeneVision N12; Mindray, China). NIRS (EGOS-600A Tissue Oxygenation Monitor; ENGINMED, China) was used to monitor SctO_2_. Sensors for NIRS were attached to the forehead with a headband that also acted as a light barrier. Noninvasive blood pressure monitoring was performed in the right arm with 1-minute interval cycles.

Basal values were obtained after 5 minutes of patient rest in the supine position. Baseline SBP and HR were defined as the means of 3 intermittent measurements with less than a 10% difference. After obtaining SBP, the levels of SctO_2_ on both sides were recorded. To reflect regional cerebral oxygenation, SctO_2_ readings from the right and left frontal lobes were averaged, and the baseline of SctO_2_ was established as the mean of 3 intermittent measurements.

Before entering the surgery room, a left-hand or forearm 18-gauge peripheral intravenous catheter was put in place. The patients were placed in the right lateral position and received a subarachnoid block. Three milliliters of 0.5% isobaric ropivacaine were injected into the estimated L3-4 or L4-5 interspace. After the subarachnoid block, patients were placed in the supine position. The level of sensory anesthesia was assessed using ice to ensure a T6 dermatomal level before the surgical incision. Patients were excluded if their sensory levels were below T6.

The patients were randomly assigned to 2 groups. Patients received either norepinephrine (8 µg/mL) or phenylephrine (100 µg/mL) at a flow rate of 30 mL/h. The infusion rate was manually controlled based on the patient’s SBP. The medication infusion was administered when the intrathecal injection was initiated and stopped after delivery. When SBP decreased by 10%, 20%, 30%, or more from the baseline, the infusion rate was increased to 40, 50, and 60 mL/h, respectively. When SBP increased by 10% or more from the baseline, we reduced the infusion rate to 10 mL/h. if patients developed hypertension (defined as an increase of ≥20% of baseline SBP), it was managed by stopping the infusion, and the infusion was restarted when SBP decreased by 10% or more from the baseline.

A noninvasive blood pressure monitor was set to cycle at 1-minute intervals until the fetus was delivered. SctO_2_ values on both sides and the HR were recorded after SBP was obtained. Eighty percent of the baseline SBP value was defined as the threshold for hypotension. If the patient had hypotension, 1 mL of “rescue drug” was given. Either 8 µg of norepinephrine was given to the NE group or 100 µg of phenylephrine was administered to the PHE group. If hypotension was not corrected within 1 minute, another rescue vasopressor bolus was given. An HR of fewer than 60 beats per minute was defined as bradycardia. If bradycardia occurred with hypotension, 0.5 mg of atropine was administered.

Mask oxygen inhalation was applied when the patient’s pulse oxygen saturation was below 95%. Ringer solution was given in doses up to 1.5 L for co-hydration. After the fetus was born, blood pressure was checked every 5 minutes, and the SctO_2_ was no longer tracked. Then, 2 U of oxytocin was given via a gradual titration.

The normalized value was defined using the following formula: Normalized value = Measured value/Baseline value × 100%.

### 2.1. Outcomes

The primary outcome in this study was the incidence of more than 10% reduction of intraoperative cerebral tissue oxygen saturation from baseline (10%ΔSctO_2_) during continuous medication infusion. Secondary outcomes included SctO_2_, maternal, fetal, and hemodynamic data (change in SBP, HR, and left- and right-SctO_2_). SctO_2_ outcomes included incidence of a more than 20% decrease in SctO_2_ from baseline (20%ΔSctO_2_), the change in SctO_2_ at 5 and 10 minutes after medication infusion, the duration of more than 10%ΔSctO_2_, and the change in nadir SctO_2_. Maternal outcomes included spinal hypotension, hypertension, bradycardia, dizziness, nausea or vomiting, intraoperative blood loss, and postoperative hemoglobin level. Foetal outcomes included the Apgar scores at 1, 5, and 10 minutes and the results of umbilical arterial blood gas analysis.

### 2.2. Statistical analysis

The sample size was calculated by detecting a more than 10% reduction of intraoperative SctO_2_ from baseline. We conducted a pilot study with 20 participants in each group and revealed that the frequency of more than 10%ΔSctO_2_ was 40% in the PHE group and 10% in the NE group. This study did not incorporate data from the pilot trial. We hypothesized that norepinephrine was more effective in maintaining SctO_2_ when preventing spinal hypotension during Caesarean birth compared with phenylephrine. The alpha error was set at 0.025 with 80% power. The sample size was calculated to be 58. Two more patients were enrolled in our study to offset potential loss to follow-up.

The Chi-squared test or Fisher exact test was used to examine categorical data, which are reported as numbers and frequencies (percentages). The Shapiro–Wilk test was used to determine the normality of continuous data, which are reported as means (standard deviation) or medians (interquartile ranges). The Mann–Whitney *U* test was used to assess continuous data that did not follow a normal distribution. The Student *t* test was used to determine continuous data with a normally distributed variable. The first 10-minute changes in SBP, HR, and left- and right-SctO_2_ data were studied. Repeated measures were analyzed using a 2-way repeated-measures analysis of variance. Statistical significance was established at a *P* value of .05. Microsoft Excel 2010 (Microsoft, Seattle, WA) was used for randomization. Data were analyzed using IBM SPSS Statistics version 20 (IBM SPSS inc., Almonk, NY).

## 3. Results

**Figure 1.**
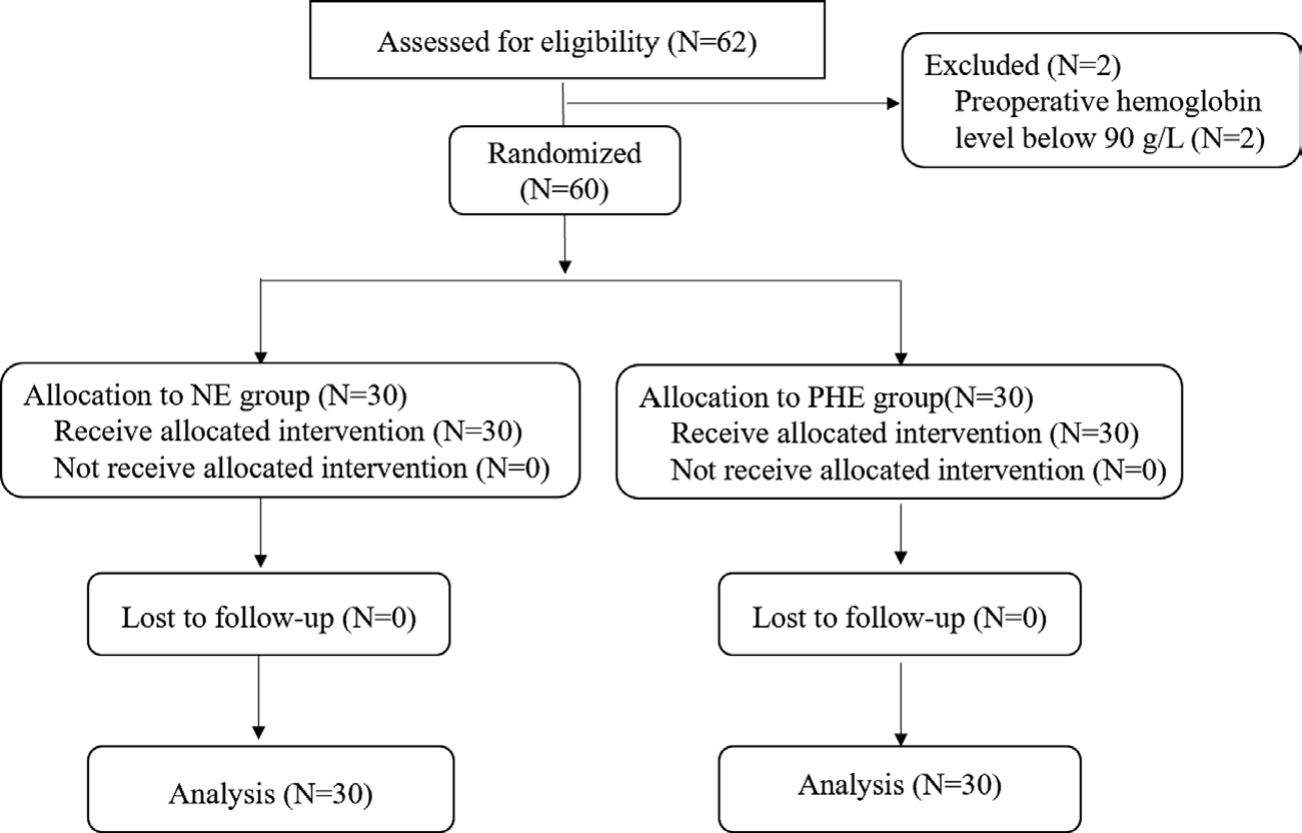
CONSORT diagram. CONSORT = Consolidated Standards of Reporting Trials, NE** **=** **norepinephrine, PHE** **=** **phenylephrine.

Sixty-two patients were enrolled in this study, with 60 patients randomized into 2 groups (30 in the norepinephrine group and 30 in the phenylephrine group; Fig. 1). Two patients were excluded because their preoperative hemoglobin levels were below 90 g/L. Table 1 displays the baseline characteristics and surgery data.

**Table 1:** Baseline characteristics and surgical data.

Characteristics	NE group (N = 30)	PHE group (N = 30)	*P* value
Age, yr	31.4 ± 3.4	30.8 ± 3.4	.45
Height, cm	162.4 ± 5.1	163.3 ± 4.6	.46
BMI, kg/m^2^	27.5 ± 3.0	28.1 ± 2.8	.41
SBP at baseline, mm Hg	117.8 ± 10.1	118.3 ± 8.1	.83
HR at baseline, beats/min	85.8 ± 9.1	88.4 ± 11.3	.31
Time from subarachnoid block to delivery, min	12.3 ± 2.0	12.1 ± 2.0	.74
Time from skin incision to delivery, min	4.8 ± 1.5	4.7 ± 1.3	.65
Preoperative hemoglobin, g/L	120.1 ± 11.5	119.9 ± 13.0	.80
ScO_2_ at baseline (right), %	63.6 ± 2.5	64.1 ± 2.4	.51
ScO_2_ at baseline (left), %	62.7 ± 2.6	63.1 ± 2.4	.44
Block-level (T)	T4 (T3.5–T5)	T4 (T3–T4.5)	.26

Data are presented as means ± SD or medians (IQR).

BMI = body mass index, HR = heart rate, IQR = interquartile range, NE** **=** **norepinephrine, PHE** **=** **phenylephrine, SBP = systolic blood pressure, SctO_2_ = cerebral tissue oxygen saturation, SD = standard deviation.

The norepinephrine group had a lower incidence of more than 10%ΔSctO_2_ than the phenylephrine group (13.3% vs 40.0%, *P* = .02, Table 2). However, there was no difference in the incidence of more than 20%ΔSctO_2_ between the 2 groups (Table 2). The change in SctO_2_ after 5 minutes of norepinephrine infusion was higher than that after phenylephrine infusion (−3.4 ± 4.7 vs −6.2 ± 5.6, *P* = .04, Table 2). The change in SctO_2_ after 10 minutes of norepinephrine infusion was higher than that after phenylephrine infusion (−2.5 ± 4.4 vs −5.4 ± 4.6, *P* = .006, Table 2). The change in nadir SctO_2_ was lower in the phenylephrine group (−8.5 ± 5.2 vs −5.4 ± 4.1, *P* = .01, Table 2). Maternal outcomes including the frequency of nausea or vomiting, dizziness, spinal hypotension, hypertension, and postoperative hemoglobin level did not show statistical differences between the 2 groups (Table 3). None of the patients needed oxygen supplementation (Table 3). Foetal outcomes were also comparable between the 2 groups (Table 4).

**Table 2:** SctO_2_ outcomes between 2 groups.

Outcomes	NE group (N = 30)	PHE group (N = 30)	OR (95% CI)	*P* value
Primary				
Incidence of more than 10%ΔSctO_2_, n (%)	4/30 (13.3)	12/30 (40.0)	0.23 (0.06–0.83)	.02
Secondary				
A more than 20% decrease in SctO_2_ from baseline, n (%)	0 (0.0)	1 (3.3)	0.97 (0.91–1.03)	.50
			Difference (95% CI)	
Duration of a 10%ΔSctO_2_, min	0 (0–0)	0 (0–2.5)	0 (0–0)	.05
Change in nadir SctO_2_, %	−5.4 ± 4.1	−8.5 ± 5.2	3.1 (0.7–5.6)	.01
Change in SctO_2_ after 5-min medication infusion, %	−3.4 ± 4.7	−6.2 ± 5.6	2.7 (0.1–5.4)	.04
Change in SctO_2_ after 10-min medication infusion, %	−2.5 ± 4.4	−5.8 ± 4.6	3.3 (1.0–5.6)	.006

Data are presented as means (SD), frequencies (%), or medians (IQR).

10%ΔSctO_2_ = 10% reduction of intraoperative cerebral tissue oxygen saturation from baseline, CI = confidence interval, IQR = interquartile range, NE** **=** **norepinephrine, OR = odds ratio, PHE** **=** **phenylephrine, SctO_2_ = cerebral tissue oxygen saturation, SD = standard deviation.

**Table 3:** Maternal outcomes.

Outcome	NE group (N = 30)	PHE group (N = 30)	OR (95% CI)	*P* value
Nausea or vomiting, n (%)	3 (10.0)	5 (16.7)	0.6 (0.12–2.6)	.70
Dizziness, n (%)	1 (3.3)	3 (10.0)	0.3 (0.03–3.2)	.61
Spinal hypotension, n (%)	5 (16.67)	6 (20.0)	0.3 (0.1–1.5)	.74
Bradycardia, n (%)	3 (10.0)	7 (23.3)	0.4 (0.1–1.5)	.15
Hypertension, n (%)	3 (10.0)	3 (10.0)	1.0 (0.22–4.6)	—
Supplemental oxygen, n (%)	0 (0)	0 (0)	—	—
			Difference (95% CI)	
Postoperative hemoglobin, g/L	112.4 ± 11.8	113.6 ± 9.9	−1.2 (−6.9 to 4.5)	.67
Intraoperative blood loss, mL	358.3 ± 92.9	396.7 ± 179.0	−38.3 (−112.1 to 35.4)	.30

Data are presented as means (SD) or frequencies (%).

CI = confidence interval, IQR = interquartile range, NE = norepinephrine, OR = odds ratio, PHE = phenylephrine, SD = standard deviation.

**Table 4:** Foetal outcomes.

Outcomes	NE group (N = 30)	PHE group (N = 30)	*P* value
PH	7.28 (7.22–7.33)	7.27 (7.23–7.32)	.44
PaO_2_, mm Hg	15 (12–18)	15 (13–18)	.74
PaCO_2_, mm Hg	50 (48–54)	51 (49–56)	.47
Apgar scores at 1 min	9 (9–10)	9 (9–10)	.34
Apgar scores at 5 min	10 (10–10)	10 (10–10)	.51
Apgar scores at 10 min	10 (10–10)	10 (10–10)	.56
Apgar scores <7 at 1 min	0 (0)	0 (0)	—

Data are presented as frequencies (%) or medians (IQR).

CI = confidence interval, IQR = interquartile range, NE** **=** **norepinephrine, PHE** **=** **phenylephrine.

**Figure 2.**
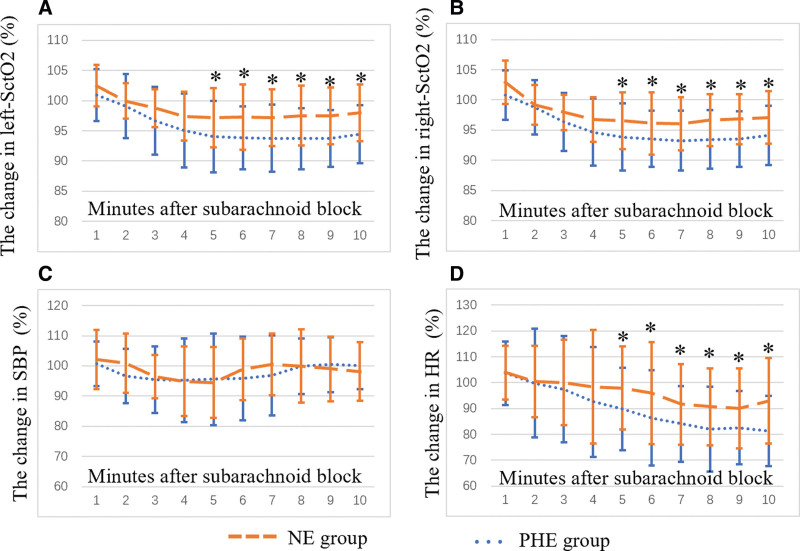
Changes in left-SctO_2_ (A), right-SctO_2_ (B), SBP (C), and HR (D). Markers are means, and error bars are SDs. The horizontal axis represents the time after medication infusion, and the vertical axis represents the changes in values. *Statistical significance between the 2 groups. HR** **=** **heart rate, NE** **=** **norepinephrine, PHE** **=** **phenylephrine, SBP** **=** **systolic blood pressure, SctO_2_** **=** **cerebral tissue oxygen saturation, SD** **=** **standard deviation.

Changes in left- and right-SctO_2_, SBP, and HR in the first 10 minutes are shown in Figure 2. The norepinephrine group showed greater left- and right-SctO_2_ values than the phenylephrine group at 5 to 10 minutes. Meanwhile, the HR was higher in the norepinephrine group than in the phenylephrine group at 5 to 10 minutes. However, the change in SBP was comparable between the 2 groups.

## 4. Discussion

This trial found that norepinephrine is more effective than phenylephrine in maintaining SctO_2_ when the prophylactic infusion was used to prevent spinal hypotension during Caesarean birth. However, the 2 medicines were equally effective in preventing spinal hypotension.

Administration of phenylephrine may lead to a reduction in SctO_2_ in anesthetized patients. A randomized trial conducted by Meng et al^[11]^ demonstrated that nonobstetric general anesthetized patients may show a 3% decrease in SctO_2_ from baseline when receiving a phenylephrine bolus. Meanwhile, Kornilov et al^[15]^ observed a reduction in SctO_2_ in patients undergoing Caesarean birth under spinal anesthesia with a 5-minute prophylactic phenylephrine infusion.^[15]^ These previous findings are consistent with the downtrend of SctO_2_ in patients with prophylactic phenylephrine infusion observed in our study. Norepinephrine is an alternative to phenylephrine in preventing spinal hypotension during Caesarean birth. However, to the best of our knowledge, data on the effect of norepinephrine on SctO_2_ during prophylactic Caesarean birth are lacking.

We found that prophylactic norepinephrine was more effective than prophylactic phenylephrine in maintaining SctO_2_ during Caesarean birth, possibly because norepinephrine is more effective at sustaining CO. Phenylephrine may cause a dose-dependent decrease in CO. Meanwhile, studies that compared norepinephrine and phenylephrine for maintaining an adequate blood pressure level during spinal anesthesia for Caesarean birth showed that norepinephrine was associated with a greater CO.^[6,16]^ Maternal HR might be an acceptable proxy for CO monitoring during spinal anesthesia for Caesarean birth.^[17]^ In our study, the norepinephrine group showed greater HR and SctO_2_ values than the phenylephrine group at 5 to 10 minutes. However, phenylephrine may cause a decrease in SctO_2_ via mechanisms other than CO modifications. Changes in SctO_2_ caused by phenylephrine infusion may be due to cerebral vasoconstriction.^[18]^

A clinically significant decrease in ScO_2_ has not yet been established. Most studies defined the theoretical cerebral hypoperfusion threshold as a decrease of more than 20% from baseline or an absolute reduction in frontal lobe SctO_2_ of more than 50% in the right or left brain.^[19,20]^ A 13% drop in ScO_2_ can be used as a threshold for cerebral ischemia with 100% sensitivity and 93.2% specificity.^[21]^ Another study by Kurihara et al^[22]^ found that in healthy individuals, a 10% to 15% reduction in SctO_2_ was associated with gravity-induced loss of consciousness. Additionally, intraoperative SctO_2_ decreased by more than 10% and was related to negative postoperative behavior changes in the pediatric population.^[23]^ A more than 10% reduction of intraoperative SctO_2_ from baseline may be associated with cerebral dysfunction or poor patient outcomes. So, we compared the incidence of 10%ΔSctO_2_ in our study.

We found that there was a significant difference in the incidence of more than 10%ΔSctO_2_ between the 2 groups, but not in the incidence of more than 20%ΔSctO_2_. The absolute difference between norepinephrine and phenylephrine may be mild and may lead to a meaningful clinical difference. Both norepinephrine and phenylephrine are effective in preventing spinal hypotension during Caesarean birth, but there were some differences in clinical outcomes, such as maternal nausea and vomiting. A Bayesian network meta-analysis of fetal and maternal outcomes conducted by Singh et al^[24]^ found that norepinephrine may be associated with a lower incidence of intraoperative nausea or vomiting than phenylephrine. Compared with phenylephrine, using norepinephrine to prevent hypotension during obstetric spinal anesthesia caused less nausea and vomiting.^[25]^ However, there is a lack of random control trials to verify the effect of both vasopressors on nausea and vomiting. Since our study had a small sample size, it was not possible to conclude.

Of note, the difference in SctO_2_ between the 2 drugs may be caused by the difference in perfusion. For mothers, the 2 drugs may also lead to different uterine or placental perfusion, especially in patients with preoperative intrauterine distress or placental hypoperfusion, leading to more pronounced benefits of norepinephrine. More studies are needed to verify the value of both drugs in critical obstetrics cases.

Our study has several limitations. First, larger and long-term outcome studies are needed to determine its clinical relevance and importance. Second, there are limitations to the routine use of NIRS as a monitor of cerebral oxygen. Extracranial blood flow affects the SctO_2_ value (signal contamination), and vasopressors may influence extracranial blood flow.^[26]^ Besides, different NIRS monitors affect the lab results. NIRS values were obtained by using various devices and computational algorithms.^[27]^ Although values obtained through different devices are not interchangeable, similar trends can be expected.^[28,29]^ Furthermore, we did not assess maternal CO because arterial puncture may cause discomfort and tension, possibly altering outcomes. Finally, the relative potency of norepinephrine:phenylephrine was determined by intermittent bolus, and may not applied for continuous infusion. Further studies need to determine the relative concentrations between the 2 drugs when applied for continuous infusion.

In conclusion, we found that norepinephrine was more effective than phenylephrine in maintaining SctO_2_ when maintaining blood pressure during spinal anesthesia for Caesarean birth. However, further studies are needed to explore the underlying mechanisms and clinical outcomes caused by SctO_2_ differences.

## Acknowledgments

The authors thank colleagues in the Department of Anaesthesiology, Qilu Hospital (Qingdao), Shandong University for their help.

## Author contributions

**Data curation:** Weiguo Wu, Qiang Zheng, Jinfeng Zhou, Xiujuan Li.

**Formal analysis:** Weiguo Wu, Haipeng Zhou.

**Investigation:** Weiguo Wu, Qiang Zheng, Jinfeng Zhou, Xiujuan Li.

**Methodology:** Weiguo Wu, Haipeng Zhou.

**Software:** Weiguo Wu, Haipeng Zhou.

**Writing—original draft:** Weiguo Wu, Haipeng Zhou.

**Writing—review & editing:** Weiguo Wu, Haipeng Zhou.

**Conceptualization:** Haipeng Zhou.
